# Long-term impact of a 4-day feed restriction at the protozoea stage on metabolic gene expressions of whiteleg shrimp (*Litopenaeus vannamei*)

**DOI:** 10.7717/peerj.8715

**Published:** 2020-03-20

**Authors:** Luis Paulo A. Lage, Delphine Weissman, Mélanie Serusier, Soraia Marques Putrino, Frederic Baron, Alain Guyonvarch, Mathieu Tournat, Alberto Jorge Pinto Nunes, Stephane Panserat

**Affiliations:** 1 INRAE, Université de Pau et des Pays de l’Adour, E2S UPPA, NuMeA, St-Pee-sur-Nivelle, France; 2 LABOMAR Instituto de Ciências do Mar / LANOA Laboratório de Nutrição de Organismos Aquáticos, Universidade Federal do Ceará, Fortaleza, Ceará, Brazil; 3 Neovia, Talhouët, Bretagne, France

**Keywords:** Feed restriction, Gene expression, Digestion, Whiteleg shrimp, Metabolism, Programming

## Abstract

Based on the “nutritional programming” concept, we evaluated the long-term effects of an early four-day caloric restriction (40% reduction in feed allowance compared to a normal feeding level) at the protozoea stage in whiteleg shrimp. We analyzed long-term programming of shrimp by studying metabolism at the molecular level, through RT-qPCR of key biomarkers (involved in intermediary metabolism and digestion). The mRNA levels (extracted from the whole body) were analyzed after the stimulus and after the rearing period, at 20 and 35 days, respectively. At the end of the experimental period, shrimp growth performance was evaluated. There was no difference between normal feed allowance (CTL) and feed-restricted shrimp (RES) for performance parameters (survival, final body weight and the number of post-larvae g^−1^ or PL g^−1^). The stimulus directly affected the mRNA levels for only two genes, i.e., *preamylase* and *lvglut* 2 which were expressed at higher levels in feed-restricted shrimp. In the long-term, higher levels of mRNAs for enzymes coding for glycolysis and ATP synthesis were also detected. This suggests a possible long-term modification of the metabolism that is linked to the stimulus at the protozoea stage. Overall, further studies are needed to improve nutritional programming in shrimp.

## Introduction

Today aquaculture has the highest growth rates among the various animal production sectors. It accounts for more than half of the global fish supply for direct human consumption as production from capture fisheries has stagnated over the last 30 years. Farm-raised marine shrimp production responds for less than 10% of the world aquaculture output and 22.6% of its total value (FAO, 2016). However, there is a growing need to improve the shrimp aquaculture to ensure its sustainability.

Several studies have shown that the whiteleg shrimp, *Litopenaeus vannamei*, can be raised with almost complete replacement of fishmeal (FM) by plant proteins, mainly soybean meal and concentrate (Suárez et al., 2009; Sookying, Davis & Soller Dias da Silva, 2013; Sabry-Neto et al., 2016). However, industrially compounded feeds continue to rely on FM as a significant source of dietary protein. Soybean meal has a lower nutrient digestibility, non-adequate amino acid profile, methionine deficiency, anti-nutritional factors and poor attractability (Gatlin et al., 2007; Tacon & Metian, 2008). However, FM is produced by the capture of wild fish, which is at risk of overexploitation. Therefore, there are ongoing efforts to develop alternative diets with a reduced reliance on FM by incorporating plant-based ingredients or byproducts obtained from agriculture and animal (Naylor et al., 2009; Nunes, Sá & Sabry-Neto, 2011; FAO, 2016). Albeit up to now, the total replacement of FM by alternative ingredients remains a difficult task, it is feasible with the supplementation of crystalline amino acids (AA) (Tacon & Metian, 2008; Nunes, Sá & Sabry-Neto, 2011). Since the development of novel diets with low levels of FM imposes several challenges, investigations into new nutritional strategies to improve the use of alternative diets become critical. In this study, we have chosen to evaluate the nutritional programming concept (Lucas, 1998; Lage et al., 2018) to better adapt shrimp to plant-based diets.

The concept of nutritional programming refers to the early events (environmental factors i.e., nutrition, toxic exposure, oxygen, temperature) performed either on the prenatal or postnatal periods which may have a persistent long-term effect either on metabolism or/and on physiology (Lucas, 1998; Patel & Srinivasan, 2002; Gluckman, Hanson & Spencer, 2005; Patel, Srinivasan & Laychock, 2009; Duque-Guimarães & Ozanne, 2013). Recently, studies on the metabolic programing in several species of fish have been successfully performed mainly focused on the early feeding (Vagner et al., 2009; Fang et al., 2014; Balasubramanian et al., 2016; Rocha et al., 2015; Rocha et al., 2016; Panserat et al., 2017).

Nevertheless, nutritional programming studies on the whiteleg shrimp *L. vannamei* are very scarce. Lage et al. (2017) mapped the genes involved in intermediary metabolism at the embryonic and larval development suggesting the existence of two developmental windows (protozoea and post-larval stages) which could be optimal to test that concept. Indeed, the first study about nutritional programming through feed restriction (well known to be a major factor of metabolic programming in mammals (Barker & Osmond, 1986; Lucas, 1998; Spencer, 2012) was performed on shrimp during the post-larvae stage. Lage et al. (2018), observed that an early nutritional stimulus improved the growth performance associated with altered mRNA levels for genes related to digestion, AA, energy, and glucose metabolism.

The present study aimed at evaluating the effect of an early feed restriction (40% lower than standard level of feeding) during the protozoea phase (between sub-stages Z1 and Z3, the second developmental window with high level of molecular plasticity (Lage et al., 2017) in order to modify the nutrient use in whiteleg shrimp, *L. vannamei*, through nutritional programming.

## Material and Methods

### Rearing conditions and shrimp larvae

Seawater was pumped from an estuary (03150001.5500S and 38125022.7400W) into 10 and 5-m^3^ reservoirs. By this time, water salinity was adjusted to the hatchery conditions (30 ups) adding fresh/tap water. This was followed by disinfection with chlorine (0.02 g L^−1^) and sand-filtering during 48 h to remove large size particles and to neutralize any chlorine residues. To assure that there was no chlorine remains in the water system, a chlorine test (Labcon CloroTest, Alcon) was performed, and finally, sodium thiosulphate (5 ppm) was added as a chlorine neutralizer.

The system was characterized by 16 rectangular tanks of 61 L in individual volume and dimensions 31.0 × 35.5 ×  55.5 cm (height ×  width ×  length), operated under a clear water recirculation system operated by a 2.0-hp water pump to sand filter (particles larger than 50 µm). Rearing tanks were connected to two reservoir tanks of 10 and 5 m^3^. In all rearing tanks, there was continuous aeration provided by two 2.0-hp air blowers and one air diffuser per tank.

One hundred and twenty thousand *L. vannamei* N3 nauplii sub-stage 3 were purchased from a commercial hatchery (CELM Aquicultura S.A., Aracati, Ceará, Brazil) and transported in plastic bags containing dissolved oxygen to the LABOMAR’s aquaculture facilities (3°50′01.55″S and 38°25′22.74″W) located in Eusebio, Brazil.

### Shrimp rearing and feeding from the early stimulus up to Day 40

**Figure 1:**
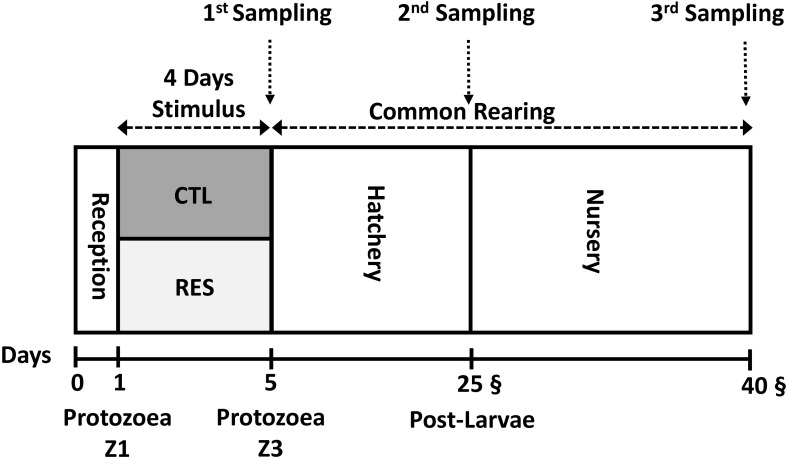
Experimental overview of the experimental design from the early nutritional stimulus at the protozoea stage up to the juvenile stage. All the samplings were done as a pool of the whole body of shrimp.

To avoid exposure to high temperature, 7,500 nauplii N3 were stocked during the nightfall period into the experimental system under and the initial density of 125 nauplii L^−1^ Fig. 1. Both hatchery and nursery phases were performed in the same rearing system. Chlorine (20 ppm) was utilized for seawater disinfection. Afterward, chlorine was neutralized with sodium thiosulphate (5 ppm).

The early nutritional stimulus started when larvae reached protozoea Z1 sub-stage 1. Animals were then divided into one experimental group (RES) and a control group (CTL) with 8 replicates tanks for each. Shrimp were fed 8 times per day following a commercial hatchery feeding guide (BernAqua NV, Olen, Belgium). Characterized using different commercial products (Table 1) was driven to the specific different stages of their development and their respective feeding habits.

**Table 1:** Stimulus Restriction Feeding Protocol for *L. vannamei* (during 4 days from protozoea Z1 sub-stage up to the Z3 sub-stage). CTL: control group. RES: feed restricted group (4% of reduction of the feeding level of the CTL group).

	Feeding Protocol (mg day^−1^)^a^		
Treatment	Day	Microalgae^b^	Royal Caviar ^c^	Vitellus Standard ^d^
CTL	1	80	10.88	
	2	80	14.88	
	3	100	18.68	
	4	120	22.28	121.50
RES	1	48	6.53	
	2	48	8.93	
	3	60	11.21	
	4	72	13.70	72.90
**Proximate composition (%)^e^**				
Protein		n.d.	55.0	51.0
Lipids		n.d.	10.0	10.0
Ash		n.d.	12.0	10.0
Fiber		n.d.	1.7	3.5
Nitrogen Free Extract^f^		n.d.	13.3	17.5
Moisture		n.d.	8.0	8.0

**Notes.**

a Feeding guide based on BernAqua NV (Olen, Belgium) recommendations.

b *Thalassiosira* spp (1,000 cel mL^−1^).

c (BernAqua NV, Olen, Belgium). Microcapsulated larval diet. Particle size from 50–100 µm.

d (BernAqua NV, Olen, Belgium). Particle size from 50–125 µm.

e Product label. Manufacturer guarantee nutrient levels.

f NFE, calculated by difference (100 –crude protein –crude fiber –crude fat –ash).

n.d., not determined.

The CTL group was fed following normal feeding rates while the treatment group (RES) was raised under a 40% restriction of the feeding level (Table 1). During the entire experiment, no disinfectant or probiotic was used. The microalgae *Thalassiosira* spp was used as a live feed from the late nauplius N5/early protozoea Z1 sub-stages to post-larvae PL3. Any nauplii *Artemia* was used at the trial and *Artemia* was completely replaced by Vitellus (BernAqua).

Before the algae inoculation in the experimental tanks; microalgae cells were determined to add the desired quantity of cell density. To feed-restricted groups, sterile saline water was added to the microalgae tank to achieve the adequate cell density, this was concerning the 40% feed restriction. The next step was that the concentration of the microalgae in the experimental tanks was determined daily before the *Thalassiosira* ssp inoculation. This process lasted until the end of the stimuli. After the early stimulus (4 days), all the experimental groups were fed similarly, following a commercial feeding protocol, until the end of the experiment on the 40th day.

### Shrimp samplings: days 5, 25 and 40

A pool of around 100 mg of shrimp larvae was sampled three different timing: after the early stimulus (Day 5), at the end of hatchery phase (Day 25), and the end of the experimental period (Day 40) (Fig. 1). For each sampling, shrimp larvae (whole body sampling) were washed with sterile saltwater and dried with an absorbent paper. Samples were preserved on an RNA Stabilization Solution (RNA Later Sigma^®^), 100 mg tissue/1 mL RNA Stabilization Solution. Subsequently, after immediate immersion in this solution, samples were kept under −20 °C.

### Total RNA extractions and relative quantification of mRNA levels

Biological material was collected with a sterile mesh (100 µm) and then dried with an absorbent paper to remove the maximum amount possible of the RNA later product. Subsequently, the sample was weighed and immediately immersed in a Trizol solution. Total RNA extractions from the whole body were performed using the reagent Trizol^®^ (100 mg sample/1 mL Trizol) and following the recommendations of the company. The total RNA concentrations were determined using the spectrophotometer NanoDrop 2000 whereas the total RNA qualities were determined after migration on a 1% agarose gel electrophoresis.

The mRNA levels were determined using the real-time RT-PCR (*n* = 6 RNA samples per treatment). The mRNA levels of 19 genes coding for proteins involved in macronutrient digestion (*lipase*, *preamylase*, *chymotrypsin*, and *trypsin*), amino acid (glutamine synthetase *gs* and glutamate dehydrogenase *gdh*), lipid (fatty acid synthase *fas*) and glucose (hexokinase *hk*, pyruvate kinase *pk*, lactate dehydrogenase *ldh*, phosphoenolpyruvate carboxykinase *pepck* and fructose 1,6-bisphosphatase *fbp*), glucose transport (*Litopenaeus vannamei* glucose transporter; *lvglut 1* and *lvglut 2*) and mitochondrial (mitochondrial cytochrome c oxidase subunit vi; *cox VI a*, *cox VI b* and *cox VI c* and mitochondrial ATP synthase subunit alpha and beta; *atpase a* and *atpase b*) metabolisms were quantified using shrimp specific primers. The primer sequences used in the real-time RT-PCR assays are the same as those used in our previous study (Lage et al., 2017).

For the RT-qPCR, an amount of 1µg total RNA was reverse transcribed to cDNA with SuperScript III RNAse H-Reverse Transcriptase Kit (Invitrogen) with random primers (Promega, USA). Real-time PCR was performed in the LightCycler 480 (ROCHE, Hercules, CA, USA). Quantitative PCR (Q-PCR) analyses for gene expressions were performed using a reaction mix of 6 µL per sample containing 2 µL of the RT product (diluted cDNA), 0.24 µL of each primer (10 µmol/L), 3 µL Light Cycler 480 SYBR^®^ Green and 0.54 µL DNase/RNase-free water (5 Prime GmbH, Hamburg, Germany). The PCR protocol was initiated at 95 °C for 10min for initial denaturation of the cDNA and hot-start enzyme activation and continued with 45 cycles of a three-step amplification program (15 s at 95 °C followed by 10 s 60 °C, for primer hybridization, and 15 s at 72 °C to extend DNA). Melting curves were systematically monitored (temperature gradient at 0.11 °C/s from 65 to 97 °C; 5 acquisitions/^∘^C) at the end of the last amplification cycle to confirm the specificity of the amplification reaction as previously described (Dai et al., 2014). Each q-PCR run included duplicates of samples (reverse transcription) and negative controls (wells without reverse transcriptase, mRNA and cDNA).

The relative quantification of mRNA levels of target genes was normalized with the *L. vannamei* elongation factor 1-alpha (*ef1a*) gene. In all cases, PCR efficiency (E) was measured by the slope of a standard curve using serial dilutions of cDNA. In all cases, PCR efficiency values ranged between 1.8 and 2.2. For the analysis of mRNA levels, relative quantification of target gene expression was performed using the Roche Applied Science *E*-Method.

### Statistical analyses

Statistical analyses were carried out using R software (v.3.1.0)/R Commander Package, shrimp performance parameters, molecular, and biochemical analyses. Before statistical analyses, the assumption of data normality and homogeneity of variances were assessed using the Shapiro–Wilk test and *Wilcoxon’s* test, respectively.

Shrimp performance, i.e., survival (%), final body weight (g) and PL g^−1^ (number of PL per gram) were compared by applying Student’s *t*-test. Data were presented as mean ±  standard deviation (SD) (*n* = 8 tanks per experimental group). Values were considered significantly different when *P* < 0.05. For the mRNA levels analysis (*n* = 6 samples per experimental group) after the stimulus and at the end of hatchery and nursery phases, statistical differences were evaluated by the Wilcoxon test as data did not follow a normal distribution. All the experimental data were presented as mean ± SD. The data were considered significantly different when *P* < 0.05.

## Results and Discussion

An important effort for an aquaculture nutritionist to understand is how to integrate the feed aspects with the physiological and digestive (metabolism) characteristics; thereby, improving the feeding management. In the shrimp farming industry, manufactured diets are typically rich in crude protein. Developing new feedings strategies such as the nutritional programming can be a useful strategy to reduce the dietary protein content and to improve the ability of the whiteleg shrimp, focusing on the use of carbohydrate without production deleterious factors.

Protozoea in shrimp is the first phase related to the exogenous feeding. As in fish (Mennigen, Skiba-Cassy & Panserat, 2013), the first feeding stage (i.e., the protozoea stage) is characterized by the strong plasticity of the metabolism at the molecular level (Mennigen, Skiba-Cassy & Panserat, 2013; Lage et al., 2017).

### Early nutritional feed restriction stimulus in protozoea shrimp: low impacts on the mRNA levels coding for metabolic and digestive proteins

The early stimulus by using a feed restriction protocol on the protozoea shrimp did not result in a decrease in shrimp performance at the end of the hatchery phase. Based on our previous study (Lage et al., 2017) we surmised that the developmental stage of protozoea (protozoea sub-stages Z1, Z2, and Z3) could be the optimal window to perform nutritional programming regarding the molecular plasticity. Hence, we chose the timing between the sub-stage Z1 and the sub-stage Z3 (4 days) to be the caloric restriction stimulus.

To test the direct effect of the early nutritional stimulus, comparison between the normal feed rate (CTL) and the one with 40% lower than normal feeding allowance (RES) (Fig. 1 and Table 1), the mRNA levels were analyzed for the genes coding for the digestion, intermediary and energetic metabolism at the whole-body level. Only *preamylase* and glucose transporter 2 (*lvglut 2*) mRNA levels were different between the conditions, in that both genes were upregulated 1.47 and 1.89 times, respectively, in the feed-restricted group, (*P* < 0.05; Table 2). The mRNA levels of *chymotrypsin* were not analyzed due to its very low level of expression as previously shown (Lage et al., 2017).

**Table 2:** Direct effect of the early feed restriction (stimulus) on the mRNA levels of the genes coding digestive, intermediary and energy metabolism measured at the whole body level of *L. vannamei* protozoea sub-stage 3 (Z3). mRNA levels were normalized by the reference gene *ef1a*. Data were presented as mean ±  SD (*n* = 6 per experimental group). CTL, control group (no feed restricted group). RES, restricted group (feed restricted group). After confirming that these data were not normally distributed assessed by Shapiro–Wilk test, statistical differences were evaluated by Wilcoxon test. Differences were considered statistically significant when *P* < 0.05. Lowercase letters indicate differences between groups. ND means no detectable gene expression.

**Stimulus**			
**Target Genes**	**CTL-history**	**RES-history**	***P*-value**
	**Means ± SD**	**Means ± SD**	
*Digestion*			
*lipase*	0.97 ± 0.28	1.17 ± 0.19	0.097
*preamylase*	0.89 ± 0.27^a^	1.31 ± 0.24^b^	**0.014**
*chymotrypsin*	ND	ND	
*trypsin*	0.95 ± 0.34	1.05 ± 0.19	0.620
*Lipid Metabolism*			
*fas*	1.11 ±. 0.55	1.07 ± 0.12	0.128
*Amino Acid Metabolism*			
*gs*	1.08 ± 0.27	1.13 ± 0.18	0.620
*gdh*	0.95 ± 0.26	0.98 ± 0.12	0.534
*Energy Metabolism*			
*atpase a*	1.06 ± 0.15	1.04 ± 0.17	0.620
*atpase b*	1.10 ± 0.20	1.14 ± 0.17	0.710
*cox VI a*	1.10 ± 0.17	1.10 ± 0.16	0.945
*cox VI b*	1.22 ± 0.28	1.13 ± 0.18	0.901
*cox VI c*	1.19 ± 0.09	1.17 ± 0.28	0.710
*Glucose Transport and Metabolism*			
*lvglut 1*	0.97 ± 0.020	1.04 ± 0.08	0.318
*lvglut 2*	0.63 ± 0.68^a^	1.13 ± 0.40^b^	**0.026**
*ldh*	1.11 ± 0.32	0.96 ± 0.16	0.137
*pk*	0.99 ± 0.15	1.04 ± 0.09	0.535
*hk*	1.01 ± 0.18	1.15 ± 0.25	0.445
*fbp*	1.09 ± 0.24	1.11 ± 0.19	0.805
*pepck*	1.00 ± 0.34	1.07 ± 0.25	0.731

Our molecular data does not suggest a strong direct effect of the stimulus on the protozoea shrimp. This could be explained by the relatively weak level of restriction (40%). Indeed, *L. vannamei* is a crustacean for which, during the early development, the species goes through typical metamorphosis (embryo, nauplius, protozoea, mysis and, post-larvae steps) before becoming juvenile and adults (Dai et al., 2014). During this process, and especially during the protozoea phase, the small size and fragility of the animals were evident. As such, imposing a stronger stimulus was not possible to avoid high mortality. Moreover, despite the role of dietary proteins (and their digestion) on the larval development (Carrillo-Farnés et al., 2007; Le Vay et al., 2001; Puello-Cruz et al., 2002), the present feeding restriction did not alter the mRNA levels of *trypsin* between CTL and RES animals. By contrast, surprisingly, *preamylase* mRNA levels were higher in RES animals. Previous studies that reported the expression of *preamylase* gene at protozoea stages (Lage et al., 2017; Wei et al., 2014b) showed strong differences between the different protozoea sub-stages (higher expression of *preamylase* gene in Z1 and Z3 stages and lower in Z2 and M1 stage. While *lvglut 2* the higher expression was observed in the protozoea Z1 sub-stage followed by a decrease to the basal level in Z2 sub-stage and furthers stages). Our data suggested that *preamylase* gene expression in RES animals could be linked to a slight modification (delay) of their development kinetic due to the dietary restriction.

Facing starvation periods or low carbohydrates as well as low protein dietary intake, the gluconeogenesis metabolism pathway is activated to improve the hemolymph glucose level avoiding metabolic dysfunctions and detrimental side effects (Cuzon et al., 2000; Rosas et al., 2001; Wang, Li & Chen, 2016). However, even though we did not observe any up-regulation of gluconeogenic pathway key enzymes (*pepck* and *fbp*), our data suggested that feed-restricted animals up-regulated the mRNA level of *lvglut 2* to increase the availability of glucose into hemolymph as a response to the lower nutrient intake.

Only *trypsin* gene expression was affected by the direct effect of feed restriction in the study performed by Lage et al. (2018, post-larvae) whereas it is not affected in the present study (Z3). The post-larvae stimulus was stronger (70%). Thus, the difference in the developmental window when the stimulus was performed as well as the strength of the stimulus could explain the differences in the reactions of the biomarkers that we observed between the two studies.

During the early development, the activity of digestive enzymes in nauplii stages for penaeid shrimp is very low. Over the metamorphoses to the late stages of protozoea up to post-larvae and the development of the digestive system, the digestive enzymes activity increased sharply as observed in protozoea to *preamylase* and *trypsin* (Muhammad et al., 2012; Wei et al., 2014b; Lage et al., 2017).

### Test of the existence of the programming through common rearing period (35 days): no modification of the shrimp performance and slight alterations of some mRNA levels coding for energy and glycolytic proteins

After the stimulus period (4 days), RES and CTL animals were reared under the same conditions until the end of the experiment on the 40th day (Fig. 1) to test the long-term effect (programming effect) of the early stimulus. We selected two samplings to test the existence of nutritional programming; the first one at the end of the hatchery phase (day 25th) and the second one at the end of the experiment (during the nursery phase) at day 40th.

Shrimp performance was analyzed on the 25th day as shown in Table 3. No significant differences between shrimp from the CTL and the RES groups were observed on the final body weight and the number of post-larvae g^−1^ or PL g^−1^. No statistical difference in the mRNA levels was observed for all the genes, except for the *trypsin* gene that was slightly up regulated in the RES group (Table 4).

**Table 3:** Performance of post-larval *L. vannamei* in the hatchery culture phase, i.e., from N3 stage up to PL12 stage (hatchery) and from N3 stage up to the PL 29 stage (juvenile; nursery). CTL, control group (no feed restriction); RES, feed-restricted group. Statistical (*n* = 8; mean ±  SD) differences was evaluated by the Student *t*-test (*P* < 0.05).

		Treatment		
	Performance	CTL-history	RES-history	P Sig.
Hatchery	Body Weight (mg)	2.14 ± 0.45	2.44 ± 1.09	0.521
	PL/g	497.21 ± 87.30	470.06 ± 46.70	0.725
Nursery	Final Survival (%)	41.00 ± 8.01	39.39 ± 5.98	0.740
	Body Weight (mg)	6.25 ± 0.65	6.40 ± 2.40	0.901
	PL/g	161.43 ± 18.10	170.06 ± 46.70	0.719

**Table 4:** Mid-term effect of the early feed restriction (programming effect) on the *mRNA* levels of the genes coding digestive, intermediary and energy metabolism of the *L. vannamei*, extracted from the whole body larvae at the end of hatchery phase (Day 25). mRNA levels were normalized by the reference gene *ef1a*. Data were presented as mean SD (*n* = 6 pools of whole body per experimental group). CTL: control group. RES, restriction group. After confirming that these data were not normally distributed assessed by Shapiro–Wilk test, statistical differences were evaluated by Wilcoxon test. Differences were considered statistically significant when *P* < 0.05. Lowercase letters indicate differences between groups. ND means no detectable.

**Hatchery**			
**Target genes**	**CTL-history**	**RES-history**	***P-value***
	**Means ± SD**	**Means ± SD**	
*Digestion*			
*lipase*	1.03 ± 0.19	0.94 ± 0.13	0.195
*preamylase*	0.95 ± 0.32	0.82 ± 0.22	0.279
*chymotrypsin*	ND	ND	
*trypsin*	0.95 ± 0.21^a^	1.10 ± 0.10^b^	**0.038**
*Lipid Metabolism*			
*fas*	0.93 ± 0.21	1.07 ± 0.29	0.328
*Amino Acid Metabolism*			
*gs*	0.98 ± 0.10	1.00 ± 0.20	0.721
*gdh*	0.77 ± 0.07	0.75 ± 0.07	0.462
*Energy Metabolism*			
*atpase a*	0.94 ± 0.08	0.98 ± 0.08	0.382
*atpase b*	1.02 ± 0.13	1.04 ± 0.11	0.916
*cox VI a*	1.03 ± 0.09	1.04 ± 0.07	0.798
*cox VI b*	0.94 ± 0.13	1.01 ± 0.07	0.127
*cox VI c*	1.06 ± 0.07	1.11 ± 0.13	0.247
*Glucose Transport and Metabolism*			
*lvglut 1*	0.89 ± 0.08	0.93 ± 0.09	0.563
*lvglut 2*	0.83 ± 0.21	0.93 ± 0.37	0.442
*ldh*	0.91 ± 0.29	1.03 ± 0.44	0.798
*pk*	0.95 ± 0.11	0.99 ± 0.19	0.752
*hk*	0.89 ± 0.17	1.03 ± 0.20	0.160
*fbp*	1.00 ± 0.16	1.06 ± 0.23	0.574
*pepck*	1.14 ± 0.69	0.88 ± 0.32	0.721

Shrimp performance was also analyzed later at the 40th rearing day which corresponded to the end of the experiment (Table 3). No significant difference between shrimp from the control and the stimulus groups was observed on survival, final body weight, and the number of post-larvae g^−1^ or PL g^−1^. We can suggest that the feed restriction stimuli were not harmful to the shrimp development as there was no difference concerning the zootechnical performance. Malnutrition in the early stages of shrimp hatchery can block the metamorphosis, essential for the hatchery process success, or produce impaired animals harming the final production process (Juarez & Moss, 2010). Furthermore, 40% feed restriction stimuli were adequate to observe mid-long-term modifications at the molecular level.

Significant differences were observed on mRNA levels for the genes coding for enzymes involved in energy metabolism *atpase a* and *b*, in anaerobic glycolysis lactate dehydrogenase (*ldh*) and aerobic glycolysis pyruvate kinase (*pk*) and hexokinase (*hk*) (Table 5). Overall, all the differentially expressed genes were up regulated on the RES group compared to the CTL group except for the *hk*. We can note these molecular biomarkers suggest that metabolic programming could be linked to the early nutritional stimulus at the protozoea phase, the first feeding stage. Studies performed with teleost fish on the nutritional programming at the early stages of the development (begging of exogenous feeding habits) have shown a long-term molecular adaptation to the genes related to the glucose metabolism (Fang et al., 2014; Geurden et al., 2014; Rocha et al., 2015). Corroborating with our findings, reinforcing the potential of the protozoea stage as the best developmental window to perform the stimulus.

**Table 5:** Long-term effect of the early feed restriction (programming effect) on the *mRNA* levels of the genes coding digestive, intermediary and energy metabolism measured at the whole body level of *L. vannamei* at the end of experiment; juvenile (PL 29). mRNA levels were normalized by the reference gene ef1a. Data were presented as mean ±  SD (*n* = 6 whole body per experimental group). CTL: control group (no feed restricted group). RES: restricted group (feed restricted group). After confirming that these data were not normally distributed assessed by Shapiro–Wilk test, statistical differences were evaluated by Wilcoxon test. Differences are statistically significant when *P* < 0.05. Lowercase letters indicate differences between groups. ND means no detectable.

**Challenge**			
**Target genes**	**CTL-history**	**RES-history**	***P-value***
	**Means ± SD**	**Means ± SD**	
*Digestion*			
*lipase*	1.23 ± 0.45	0.94 ± 0.39	0.234
*preamylase*	1.28 ± 0.28	1.07 ± 0.36	0.234
*chymotrypsin*	1.02 ± 0.18	1.10 ± 0.62	0.878
*trypsin*	1.17 ± 0.64	1.05 ± 0.56	0.878
*Lipid Metabolism*			
*fas*	1.14 ± 0.29	1.05 ± 0.18	0.442
*Amino Acid Metabolism*			
*gs*	1.24 ± 0.35	1.09 ± 0.25	0.574
*gdh*	1.02 ± 0.15	1.18 ± 0.23	0.105
*Energy Metabolism*			
*atpase a*	1.00 ± 0.09^a^	1.18 ± 0.16^b^	**0.028**
*atpase b*	1.04 ± 0.10^a^	1.30 ± 0.23^b^	**0.049**
*cox VI a*	1.16 ± 0.18	1.24 ± 0.28	0.574
*cox VI b*	1.16 ± 0.07	1.09 ± 0.23	0.721
*cox VI c*	1.07 ± 0.08	1.25 ± 0.25	0.105
*Glucose Transport and Metabolism*			
*lvglut 1*	1.07 ± 0.20	1.14 ± 0.02	0.505
*lvglut 2*	1.25 ± 0.41	0.96 ± 0.34	0.234
*ldh*	0.90 ± 0.12^a^	1.36 ± 0.44^b^	**0.038**
*pk*	1.00 ± 0.18^a^	1.51 ± 0.38^b^	**0.015**
*hk*	1.28 ± 0.25^b^	1.06 ± 0.19^a^	**0.049**
*fbp*	1.15 ± 0.16	1.19 ± 0.15	0.955
*pepck*	1.12 ± 9.16	0.98 ± 0.30	0.574

However, these significant differences are relatively weak (low level of statistical significance), and there was a lower number of differentially expressed genes by programming if compared with our previous study (Lage et al., 2018). This could be explained by the strong differences between the two studies of nutritional programming in shrimp (the present study and the one performed by (Lage et al., 2018): the different levels of the dietary restriction (40% versus 70%), the distinct developmental windows used (protozoea versus post-larvae), the duration of the full experiment (45 days versus 127 days), and a dietary challenge used only in the first study.

## Conclusion

The data shown above, indicates that an early nutritional stimulus (at the level of 40% feeding restriction) for the protozoa stage was not able to change permanently or significantly enough, the metabolism and growth performance in shrimp. We also observed that medium to long term modification on genes were mainly related to glucose and energy metabolism. Thus, we can state that those biomarkers are appropriate tools to evaluate the nutritional programming in whiteleg shrimp through the feed restriction. Further studies are thus needed to improve our knowledge about the possibility of the use of programming shrimp for better growth performance and nutrition in aquaculture.

##  Supplemental Information

10.7717/peerj.8715/supp-1 Supplemental Information 1 RT-qPCR raw data related to the feeding restriction effect

10.7717/peerj.8715/supp-2 Supplemental Information 2 RT-qPCR raw data measured at the end of hatchery phase (25th day)

10.7717/peerj.8715/supp-3 Supplemental Information 3 RT-qPCR raw data measured at the end of experiment, long-term effect of the early feeding restriction (40th day)

## Additional Information and Declarations

### Data Availability

The following information was supplied regarding data availability: The results of the gene expression studies are available in the Supplementary Files.
